# Towards shot-noise limited diffraction experiments with table-top femtosecond hard x-ray sources

**DOI:** 10.1063/1.4991355

**Published:** 2017-07-19

**Authors:** Marcel Holtz, Christoph Hauf, Jannick Weisshaupt, Antonio-Andres Hernandez Salvador, Michael Woerner, Thomas Elsaesser

**Affiliations:** Max-Born-Institut für Nichtlineare Optik und Kurzzeitspektroskopie, 12489 Berlin, Germany

## Abstract

Table-top laser-driven hard x-ray sources with kilohertz repetition rates are an attractive alternative to large-scale accelerator-based systems and have found widespread applications in x-ray studies of ultrafast structural dynamics. Hard x-ray pulses of 100 fs duration have been generated at the Cu K_*α*_ wavelength with a photon flux of up to 10^9^ photons per pulse into the full solid angle, perfectly synchronized to the sub-100-fs optical pulses from the driving laser system. Based on spontaneous x-ray emission, such sources display a particular noise behavior which impacts the sensitivity of x-ray diffraction experiments. We present a detailed analysis of the photon statistics and temporal fluctuations of the x-ray flux, together with experimental strategies to optimize the sensitivity of optical pump/x-ray probe experiments. We demonstrate measurements close to the shot-noise limit of the x-ray source.

## I. INTRODUCTION

The precise determination of the ground state electron density *ρ*(**r**) of matter in the solid state by x-ray diffraction and its analysis have developed into a mature field and provide detailed insights into the nature of chemical bonding in a great variety of materials.^1,2^ The study of transient electron density *ρ*(**r**, *t*), gained from femtosecond x-ray diffraction employing an optical pump/x-ray-probe setup, is an exciting recent development. It allows for atomic motion or relocation of electronic charge, both relevant in chemical or physical processes, to be resolved on atomic time and length scales.^3–11^ The required femtosecond hard x-ray pulses have been generated by accelerator-based sources, such as free electron lasers or slicing schemes at synchrotrons, and by laser-driven table-top femtosecond hard x-ray sources. The latter offer a moderate hard x-ray flux with a negligible timing jitter between pump and probe pulses, long-term access for lab-based experiments, and comparably low cost for implementation.^12–17^ The advent of laser-driven sources with kilohertz repetition rates has strongly enhanced the experimental sensitivity, and intensity changes as small as $\Delta I/I_{0}=(I-I_{0})/I_{0}=10^{-3}$ (*I* and *I*_0_: intensity diffracted with and without excitation of the sample, respectively) have been measured in femtosecond powder diffraction experiments.^7^

The generation of femtosecond hard x-ray pulses in a laser-driven source is based on vacuum acceleration of electrons on the time scale of the optical cycle of the driving laser pulse, innershell ionization of metal atoms in the target, and subsequent spontaneous and entirely uncorrelated emission of characteristic x-ray photons.^18,19^ The time structure of the generated x-ray pulses is determined by the duration of the driving pulses and the transit time of decelerating electrons through the thin metal target. An X-ray pulse duration of 100 fs has been demonstrated with sub-50 fs driver pulses centered at 800 nm and a sub-20 *μ*m target thickness. The application of sub-100 fs pulses centered at 3.9 *μ*m has allowed for generating up to 10^9^ hard x-ray photons per pulse, an increase by a factor of 20 compared to a 800 nm driver.^20^ The generated x-ray flux displays fluctuations on various time scales, requiring efficient normalization and averaging methods to measure small changes in the diffracted intensity. While these issues require an optimization of the experimental parameters, a fundamental limit consists in the photon counting statistics.

In this article, we analyze the photon statistics of x-ray pulses from a femtosecond laser-driven hard x-ray source and various mechanisms behind intensity fluctuations. Different schemes to mitigate the influence of fluctuations in ultrafast diffraction experiments are considered. We then concentrate on a particular pump-probe setup and demonstrate nearly shot-noise limited time-resolved diffraction experiments with single crystalline LiNbO_3_ and Bi samples. The results provide deeper insights into the characteristic spatial and temporal fluctuations of the hard x-ray source.

## II. EXPERIMENT

**FIG. 1.**
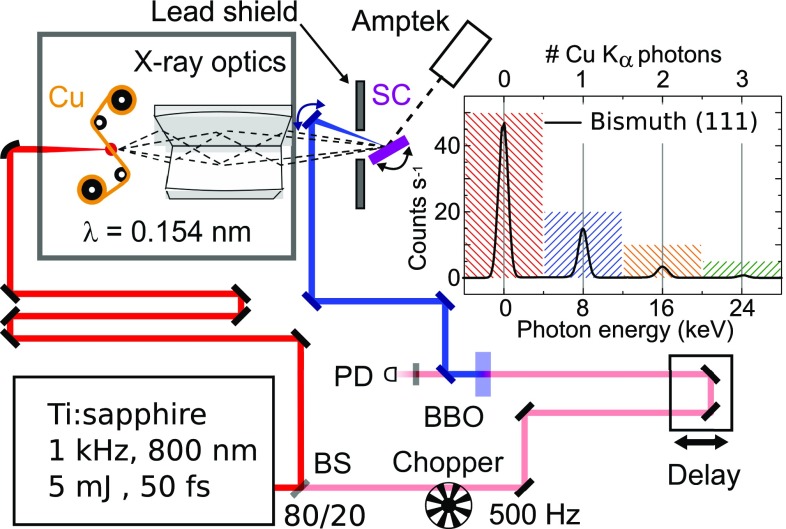
Schematic drawing of the pump-probe setup employed for the femtosecond diffraction experiment on single crystals (SC). The samples are excited by 400 nm pulses generated by frequency doubling in a 250-*μ*m-thick BBO (type I phase-matching). The pump arm is mechanically chopped with 500 Hz, and a photodiode (PD) determines at 1 kHz, i.e., for every x-ray probe pulse, if the sample was excited. The number of x-ray photons that are diffracted from the sample is determined with an energy-resolving CdTe diode (Amptek). The inset shows a spectrum with 0.4 diffracted photons per pulse, i.e., a case of a very low x-ray flux on the detector.

The ultrafast diffraction experiments are based on the pump-probe scheme illustrated in Fig. 1. The sample is optically excited by femtosecond pulses with a center wavelength of 400 nm and probed with hard x-ray pulses. Both pump and x-ray probe pulses are derived from an amplified Ti:sapphire laser system delivering sub-50 fs pulses centered at 800 nm with an energy of 5 mJ and a repetition rate of 1 kHz. The major part (80%) of the 800 nm laser output is focused to a spot size of approximately 10 *μ*m on a 20 *μ*m thin Cu tape. The tape is moved electromechanically with a speed of some 0.05 m/s to provide a fresh target volume for each laser pulse. Plastic tapes moving in parallel collect the debris from the Cu target. In this geometry, hard x-ray pulses with a photon energy of 8.04 keV (Cu K_*α*_) and a duration of roughly 100 fs are generated. The emitted x-ray pulses are collected, monochromatized, and focused onto the sample by a Montel multilayer mirror (Incoatec). The Cu K_*α*_ flux on the sample has a value of approximately ∼5 × 10^6^ photons/s. Other technical details of the setup have been reported in Ref. 17.

In the present experiments, individual Bragg peaks from single crystalline LiNbO_3_ (Ref. 21) and Bi samples^4,10^ were recorded in reflection. The x-ray photons were detected with a CdTe diode (Amptek). This detector displays a quantum efficiency of nearly 100% for Cu K_*α*_ photons and is energy-resolving. It features a large active area of 9 mm^2^ and can be read out with a repetition rate of 1 kHz of the laser system, i.e., negligible dead-time. The inset in Fig. 1 shows an exemplary spectrum of 5.3 × 10^6^ x-ray shots with an average of 0.4 diffracted photons per shot. Here, the reflectivity on the (111) Bragg reflection from a 40 nm thick Bi film grown epitaxially on a silicon (111) wafer was determined. Events with 0, 1, 2,… detected photons are clearly distinguished by their corresponding energies of 0, 8, 16,… keV within the color coded areas. These features allow for determining the number of diffracted photons on an individual Bragg reflection for each x-ray pulse individually.

In the case of polycrystalline samples, a large area detector (Pilatus Dectris 1M) has been used to record the diffracted hard x-ray photons of multiple Debye-Scherrer rings simultaneously in a transmission geometry.^22^ Since the maximum readout rate of this detector is 30 Hz, the detector is triggered to accumulate x-ray photons diffracted from 37 sequential pulses in a single exposure, followed by an additional dead-time of 3 ms for the detector readout. This detector is not truly energy-resolving but allows for setting a lower energy limit for detection. Therefore, one has to ensure that each pixel of the detector (172 × 172 *μ*m^2^) is hit by only one photon during a single exposure to enable photon counting. This criterion is typically fulfilled when polycrystalline samples are studied, due to their much lower scattering efficiency per solid angle.

## III. RESULTS AND DISCUSSION

### A. Photon statistics and fluctuations of x-ray flux

Laser-driven hard x-ray sources are characterized by a strongly varying number of generated x-ray photons over time. In the case of an ideal x-ray source with a constant average x-ray flux $\bar{n}$, the number of photons per shot k would obey the Poisson distribution, as defined by^23^

$$P_{\bar{n}}^{\text{pois}.}\;(k)=\frac{{\bar{n}}^{k}}{k!}\text{exp}\;(-\bar{n}).$$ (1)

Such an ideal source possesses only white noise since the fluctuations of the number of x-ray photons are solely caused by the intrinsic shot-noise. The experimental uncertainty in counting x-ray photons in this case would be determined by the total number of measured photons. A different kind of source, which features temporal fluctuations in addition to the shot-noise, is a chaotic light source, where the number of photons per shot obeys a Bose distribution

$$P_{\bar{n}}^{\text{chao}.}\;(k)=\frac{{\bar{n}}^{k}}{{\left(1+\bar{n}\right)}^{\left(1+k\right)}}.$$ (2)

**FIG. 2.**
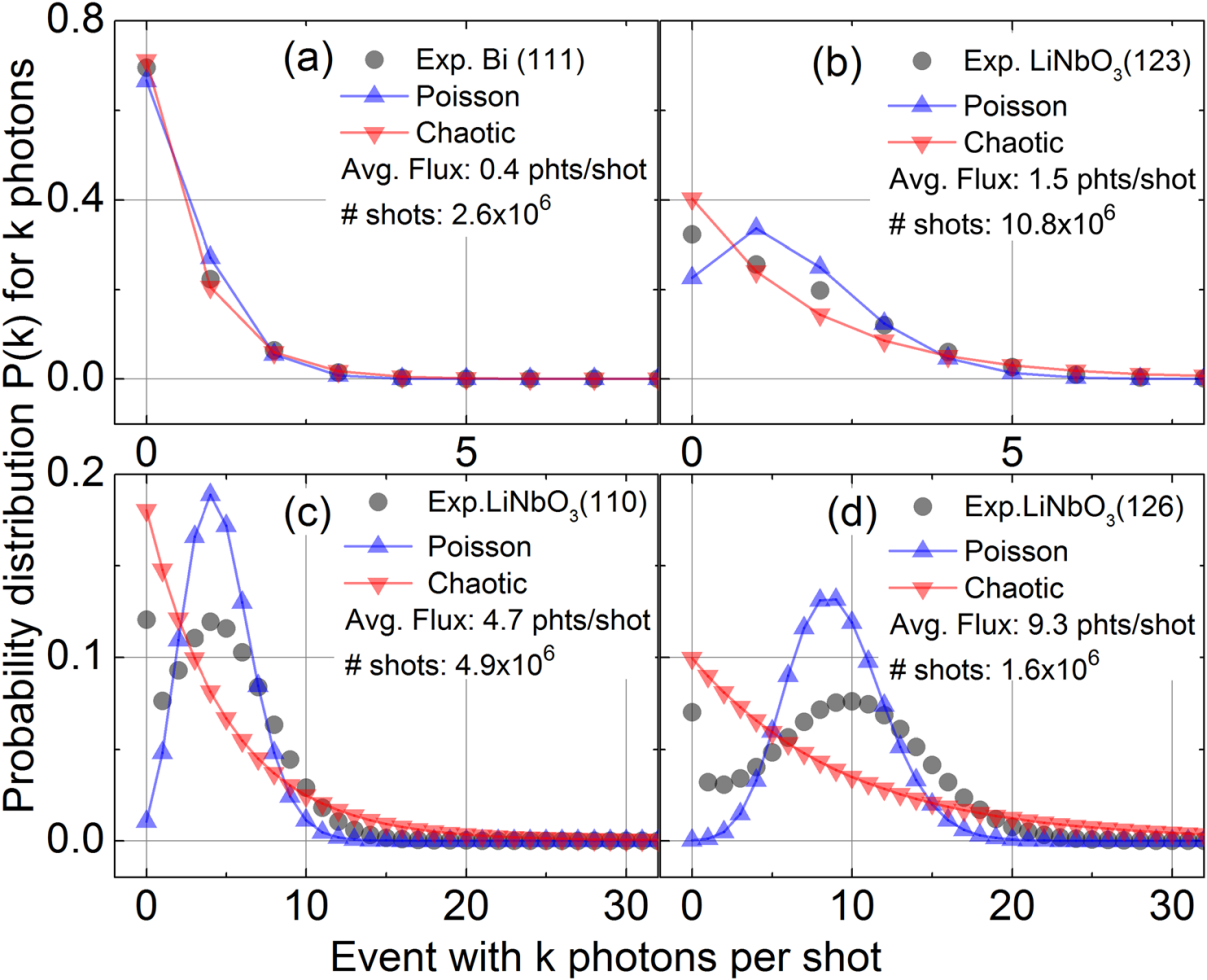
Probability distributions for k photons being detected by the energy-resolved x-ray detector for different average fluxes. Grey symbols represent experimental results, while blue and red symbols represent simulated values based on the same average flux, assuming an x-ray source with purely Poissonian or chaotic characteristics. (a) Results for the (111) Bragg reflection of a thin Bi film with an average flux of 0.4 photons/shot. The three distributions are very similar, in this case with a photon flux less than one. The results in panels (b)–(d) were obtained on the (123), (110), and (126) Bragg reflections of an X-cut LiNbO_3_ single crystal. Here, the differences between the three distributions become apparent, as the flux is significantly larger than in the case of the Bi (111) Bragg reflection. The experimentally observed distribution is neither purely chaotic nor purely Poissonian. It shows characteristics of both distributions, i.e., the non-vanishing probability at low photon numbers as in chaotic light and a clear maximum as in a Poisson distribution.

Figure 2 shows the calculated probability distribution for 0, 1, 2,… photon events being recorded per shot from an ideal light source (blue symbols) and a chaotic light source (red symbols) for four scenarios with different average fluxes. Such predictions are compared to experimental data (grey symbols) recorded with the CdTe detector on a shot-to-shot basis. The total number of shots recorded is given in the insets of the panels, and the experimental uncertainty of the probability values is less than the size of the symbols. For a low number of detected photons per shot (≤0.5 photons/shot), the two theoretical probability distributions are almost indistinguishable from each other [Fig. 2(a)] and fit nicely with the experimental values measured with the Bi sample. For a higher flux of x-ray photons diffracted from the LiNbO_3_ crystal for the same incoming x-ray flux [Figs. 2(b)–2(d)], one observes a systematic deviation from a pure Poisson character. It should be noted that the four different photon distributions do not contain any microscopic information about either the x-ray generation process or the state of the excited sample. The different Bragg reflections have solely been chosen as representative examples with different average fluxes. In these cases, the measured data can be best described as an intermediate case between pure Poisson light and a slight admixture of chaotic light. These differences to an ideal x-ray source gradually become more pronounced with increasing average flux and are caused by additional fluctuations of the generated flux. Without appropriate countermeasures, these fluctuations increase the experimental error quite significantly.

**FIG. 3.**
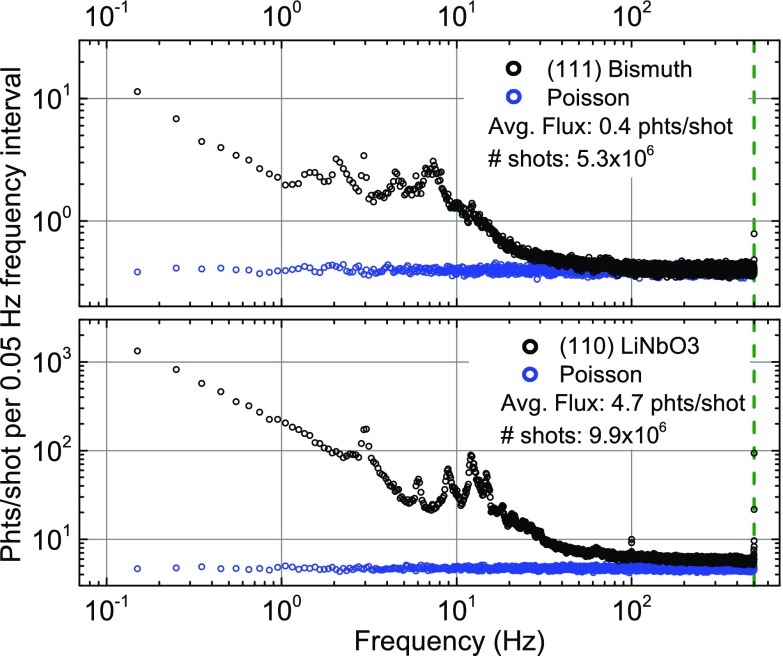
Power spectrum of the x-ray photon flux diffracted from (a) a Bi and (b) a LiNbO_3_ crystal, in comparison to the spectrum of a source with a Poisson photon statistics with the same average flux. Each point is averaged over a frequency interval of 0.05 Hz. The experimental spectra display distinct frequency components which originate from different mechanisms discussed in the text.

The laser-driven x-ray source displays intensity fluctuations on different time scales. For an in-depth characterization, we determined the frequency spectrum of the fluctuations by analyzing the photon flux diffracted on the (111) Bragg reflection from the Bi sample and on the (110) reflection from the LiNbO_3_ crystal. The resulting power spectra are presented in Fig. 3. Additionally, the blue symbols represent the spectra of a source with the same average flux that exhibits pure Poisson photon statistics and therefore displays only white noise. Both experimental spectra exhibit a smooth decrease by two orders of magnitude in amplitude between 0.1 and 100 Hz that roughly obeys an f^−1^ dependency on the frequency f, indicative of a source exhibiting fluctuations with the overall characteristics of pink noise. This is superimposed by comparably narrow features between 1 and 30 Hz. The high fluctuation amplitude at 0.1 Hz and below (not shown) is due to slow nonperiodic fluctuations on a time scale from tens of seconds to hours. Here, residual mechanical instabilities of the setup, slow drifts in the focusing conditions of the laser pulses on the target, and a small continuous drift of the target position due to a variation of mechanical tension in the moving copper tape are relevant.

In the frequency range between 1 and 100 Hz corresponding to a time scale from 10 ms to 1 s, the spooling mechanism of the target tape introduces distinct fluctuation components. Even small mechanical displacements from the ideal target position by some 50 *μ*m will reduce the x-ray flux by an order of magnitude.^17^ This periodic mechanism gives rise to the spikes in the spectra shown in Fig. 3, occurring at the characteristic angular speed of different components of the spooling system. Mechanical instabilities of the plastic tape, which runs in parallel to the copper tape target and serves for debris protection,^17^ contribute to such fluctuations as well. The minute differences between the two experimental spectra seen in this frequency region are simply due to small variations of the day to day performance of the spooling mechanism.

Even for frequencies of more than 100 Hz, the experimental fluctuation spectra and the corresponding Poisson distribution differ, demonstrating the occurrence of fluctuations of x-ray flux on comparably short time scales. This is particularly evident in the second spectrum, which has been measured with a higher average flux. Mechanisms contributing to this are a variation of the granularity of the metal target and shot-to-shot fluctuations of the pulse duration and/or pulse energy of the driving 800 nm laser pulses. Shot-to-shot fluctuations also arise from the interaction of the generated x-rays with single crystalline domains of the remaining copper target surrounding the very small pumped volume of the target, leading to a non-isotropic emittance of Cu K_*α*_ photons.^24,25^ Even for a constant number of x-ray photons emitted in the entire solid angle per shot, such a scenario leads to temporal changes in the x-ray flux on the sample since the x-ray optics only collects photons from a finite solid angle.

### B. Enhancement of experimental sensitivity

Ultrafast x-ray diffraction experiments based on pump-probe schemes measure pump-induced changes in the x-ray flux diffracted from the probe pulse on particular Bragg reflections and/or Debye-Scherrer rings. To enhance the experimental sensitivity, i.e., measure relative intensity changes as small as possible, different schemes for normalizing the x-ray flux have been introduced. Most of them are based on a reference x-ray flux derived from the same source and detected with a second detector.^26–31^

**FIG. 4.**
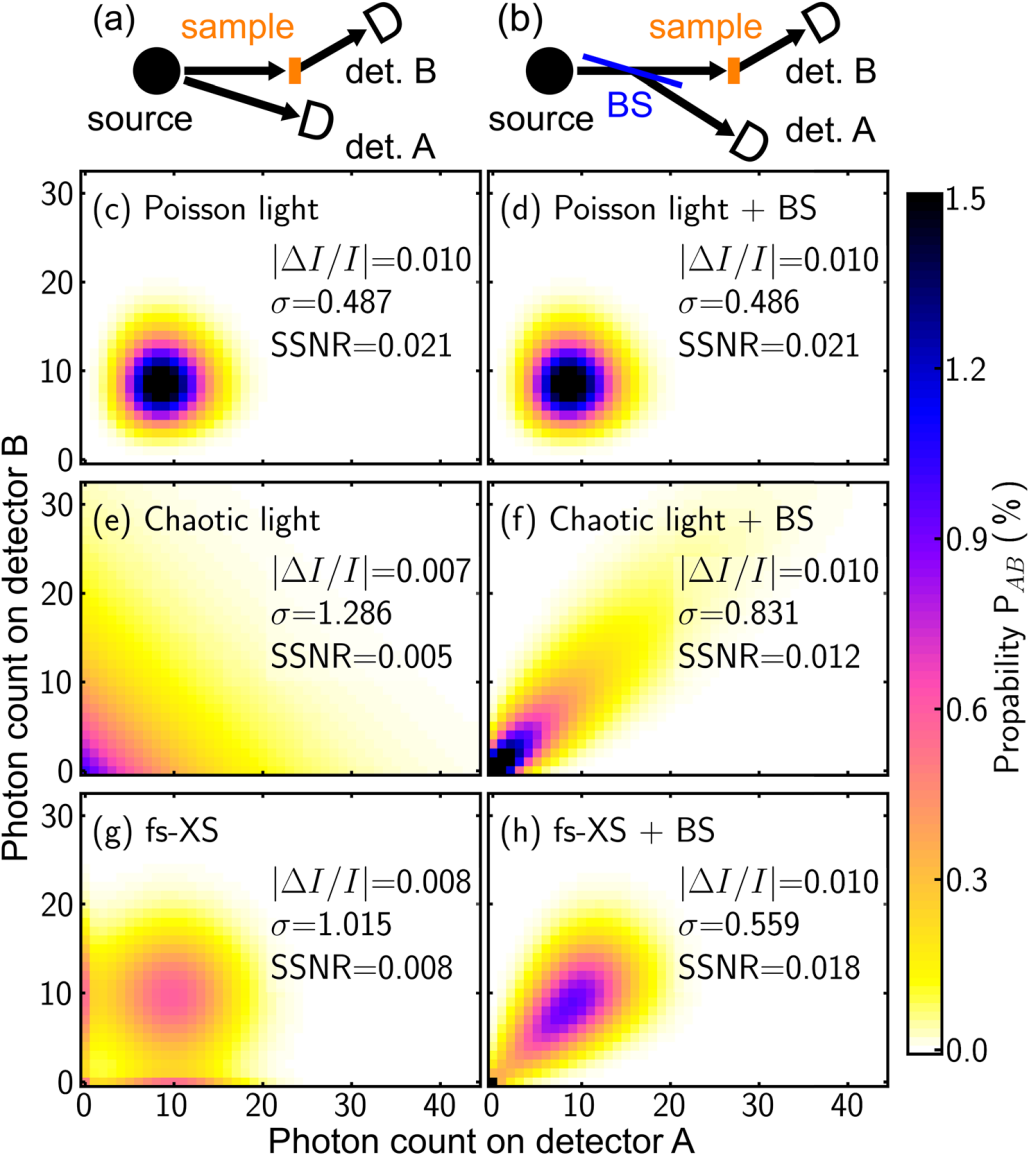
Illustration of two fundamental normalization concepts, with a direct observation of the source (a) or the ideal (50/50) beamsplitter (b) and a sample with a reflectivity of 99%. (c)–(h) Comparison of the probability distribution functions (PDF) for three sources with an identical average flux but different types of statistical properties [Poisson (top), chaotic (middle), and real fs x-ray source (bottom)] for the two normalization concepts. The resulting PDFs for detecting an event with a certain photon number on the reference detector A (x-axis) and detector B (y-axis) are presented by a color coded scale, and the recovered change of diffraction intensity Δ*I*/*I* according to Eq. (3), the corresponding error *σ*, and the resulting single shot signal-to-noise ratio (SSNR) is given for each case.

In this section, we consider two basic normalization concepts, the detection of an independent reference flux from the same source [Fig. 4(a)] and the introduction of a beam splitter dividing the incoming x-ray flux into a first part interacting with the sample and a second part serving as a reference [Fig. 4(b)]. Our analysis is based on the following assumptions:

(i) There are two identical detectors A (reference) and B (signal) which detect the same average number $n_{A}=n_{B}^{0}=9$ of x-ray photons per shot in case the sample is not excited by the pump pulse.
(ii) With an ideal (50/50) x-ray beamsplitter, each incoming x-ray photon has an equal probability to be sent into either the signal or the reference beam path.
(iii) Upon excitation of the sample, the photon flux is reduced by one percent, i.e., $n_{B}=0.99n_{B}^{0}$.
(iv) The normalized pump-probe signal is given by
  $$\frac{\Delta I}{I}=\frac{n_{B}-n_{B}^{0}}{n_{B}^{0}}\approx 2\cdot \frac{n_{B}-n_{A}}{n_{B}+n_{A}}.$$ (3)

In our case, the signal has a value of |ΔI/I|=0.01. For our analysis, we use the last expression in Eq. (3), where $n_{B}^{0}$ in the denominator has been replaced by n_*B*_. The resulting systematic error is tolerable as long as the intensity change is below a few percent.

In Figs. 4(c)–4(h), we present a comparison of results for three types of femtosecond x-ray sources with an identical average flux $\bar{n}$, but different statistical properties [Poisson (c)–(d), chaotic (e)–(f), and real fs x-ray source (g)–(h)]. The results for the direct normalization scheme (left column) are compared to the geometry with a beamsplitter (right column). The probability distribution functions (PDF) of detecting *n*_A_ and *n*_B_ x-ray photons on detector A (x-axis) and detector B (y-axis) for the same shot are plotted in a color coded 2D plot for each of the six different cases. In the case of the direct normalization scheme, the corresponding probability distribution functions $P_{A,B}^{\text{no}\;\text{BS}}(n_{A},n_{B})$ are given by the direct product of the distributions P_A_(n_A_) and P_B_(n_B_) of the two separate detectors

$$P_{A,B}^{\text{no}\;\text{BS}}(n_{A},n_{B})=P_{A}(n_{A})\cdot P_{B}(n_{B}).$$ (4)

In the case of a beamsplitter, the corresponding probability distribution function $P_{A,B}^{\text{BS}}(n_{A},n_{B})$ is given by^23^

$$P_{A,B}^{\;\text{BS}}(n_{A},n_{B})=P(n_{A}+n_{B})\;\frac{(n_{A}+n_{B})!}{n_{A}!\;\;n_{B}!}\;{\left|R\right|}^{n_{A}}\;{\left|T\right|}^{n_{B}}.$$ (5)

Here, P(n_A_ + n_B_) is the incoming distribution function, while the remaining part describes a binomial distribution, where R and T are the (intensity) reflectivity and transmittivity of the beamsplitter; in our case, R = T = 0.5.

Figures 4(c) and 4(d) compare both methods for an x-ray source with purely Poissonian statistics of photons per shot according to Eq. (1). In this case, the value of |ΔI/I|=0.01 is well recovered, coupled with an error which is only determined by the intensity fluctuations due to the shot-noise limit for both normalization methods. Therefore, both cases display equally good results and yield a single shot signal-to-noise ratio (SSNR) of 0.021. Figures 4(e) and 4(f) show $P_{A,B}(n_{A},n_{B})$ for a fully chaotic light source containing additional intensity fluctuation, as described by Eq. (2). The direct normalization results in a SSNR is about ∼4 smaller in comparison to pure Poisson light, mainly caused by the increased error (∼2.5 times higher), since both detectors measure fully uncorrelated flux. Using a beam splitting scheme in combination with a chaotic light source leads to a correlated measurement on both detectors, which is indicated by the reduced width of $P_{A,B}(n_{A},n_{B})$ along the diagonal. Therefore, the SSNR can be enhanced by approximately a factor of 2 in this case.

Examples derived from measurements with the LiNbO_3_ crystal employing the femtosecond x-ray source that provided the same average x-ray flux in this case are shown in Figs. 4(g) and 4(h). The shape of the probability distribution $P_{A,B}(n_{A},n_{B})$ matches neither the fully chaotic nor the Poisson distribution in this scenario. The direct normalization for the table-top source provides a SSNR that is only marginally better than in the case of a fully chaotic light source, and is therefore not the ideal choice for a proper normalization. This problem would be even more pronounced if the emittance of the x-ray source were to exhibit spatially anisotropic characteristics (see above). Compared to the direct normalization, the introduction of a beamsplitter approximately doubles the SSNR, which in this case almost reaches the value of an x-ray source with purely Poissonian photon flux.

**FIG. 5.**
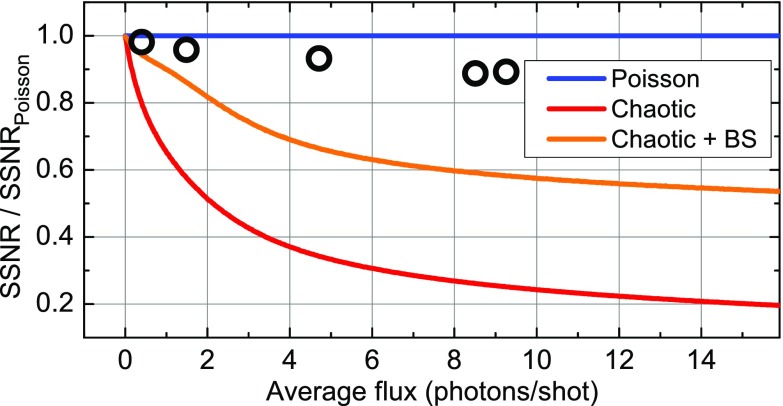
The single shot signal-to-noise ratio (SSNR) normalized to the SSNR for a Poisson photon statistics as a function of the average x-ray flux. The results are presented for different cases in Fig. 4, together with points from different data sets (open black circles).

Figure 5 shows ΔSSNR = SSNR/SSNR_poisson_, i.e., the dependence of the recoverable SSNR for different cases discussed in Fig. 4 on the average flux, normalized to the SSNR_poisson_ for pure Poisson light. As shown above, the direct normalization scheme and a beamsplitter yield identical results in the latter case and are thus represented by a single blue line. Experimental results are shown as open black circles. For a decreasing average flux, the character of both the fully chaotic light source and the table-top source increasingly resemble the pure Poisson case, and a (nearly) shot-noise limited measurement is possible in all five cases. This result allows for designing an optimization strategy for the experimental sensitivity. The combination of a high average x-ray flux from the laser-driven source with a diffracted x-ray flux at the level of few photons per shot provides the highest signal-to-noise ratio (SNR) in the measurements. This fact clearly favors laser drivers with repetition rates in the kilo- to megahertz range. A promising ongoing driver development based on optical parametric chirped pulse amplification (OPCPA) combines a mid-infrared center wavelength of the driving pulses with a repetition rate of up to 10 kHz and an optical average power up to tens of watts.^20,32^

The design of a practical x-ray beamsplitter scheme fulfilling the conditions discussed so far has remained difficult.^33^ However, an equivalent, even simpler solution consists in mechanically chopping the pump pulses in the pump-probe setup and detecting the x-ray flux diffracted in subsequent events with and without pump with a single detector. Here, the chopping frequency has to be chosen in accordance with the detector used to record the x-ray photons. The CdTe diode used in the case of single crystalline samples can be read out faster than the 1 kHz repetition rate of the laser system, and thus, a chopping frequency of 500 Hz allows for measuring the x-ray signal from the excited/unexcited sample on a shot-to-shot basis. This scheme requires a proper synchronization by an independent detection of a small fraction of the chopped pump beam [detector photodiode (PD) in Fig. 1].

**FIG. 6.**
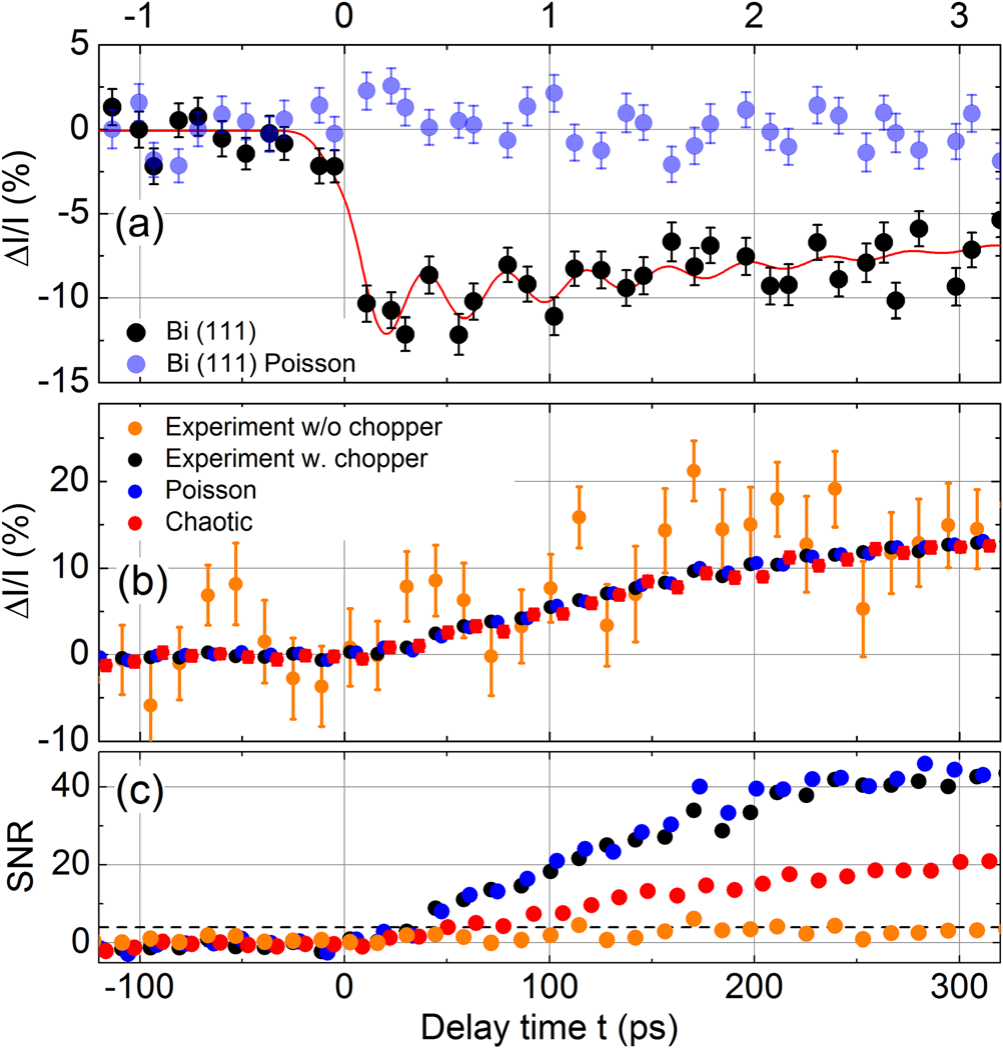
(a) The transient change in diffracted intensity Δ*I*/*I* as a function of the pump-probe delay for the (111) Bragg reflection of a 40 nm thin Bismuth film upon excitation with 400 nm pump-light. The black symbols represent the actual experimental results. Additionally, the light blue symbols correspond to an ideal Poisson-distributed x-ray source with constant and delay independent flux and the same number of photons detected as in the experiment. (b) Transient change in diffracted intensity Δ*I*/*I* as a function of the pump-probe delay for the (110) Bragg reflection of an X-cut LiNbO_3_ single crystal. The black symbols represent the actual experimental results, while the dark blue and red symbols depict simulated transients assuming an x-ray source with purely Poissonian or chaotic characteristics, respectively. The orange symbols represent experimental data, in which the influence of the mechanical chopper has been removed. (c) The corresponding SNR for different curves shown.

Normalization by chopping the pump pulse sequence allows for measuring small transient intensity changes with a time resolution of ∼100 fs and a sufficient SNR in a comparably short data acquisition period. As an example, we present in Fig. 6(a) the time evolution of the change in diffracted intensity Δ*I*/*I* on the (111) Bragg reflection of a single crystalline 40 nm thick bismuth film upon excitation with 400 nm pump pulses (excitation fluence ≈ 1.2 mJ cm^−2^; pulse duration ≈ 70 fs). In this experiment, we have employed the so-called “rapid-scanning technique,” where a total of 1050 individual delay times with a short integration time of 5 s per delay time were recorded in random order over the course of the total experiment time of 90 min and then averaged. The time dependent change in diffracted intensity Δ*I*/*I* is given by Eq. (3), requiring individual events with a non-zero denominator. This is ensured by stacking pairs of sequentially measured pumped/unpumped shots together, when deriving the average signal.

The black symbols in Fig. 6 represent the time dependent change in diffracted intensity Δ*I*/*I* on the Bi (111) Bragg reflection, which agrees well with previously published results.^4,34^ This clearly documents that the present experimental protocol allows for performing ultrafast diffraction experiments with a 100 fs time resolution and a SNR of up to 12 in a reasonable data acquisition period. The significant improvement of the SNR that is achieved by introducing a mechanical chopper as a normalization scheme is evident from the transients in Fig. 6(b), where the time dependent change in diffracted intensity Δ*I*/*I* on the (110) Bragg reflection of the LiNbO_3_ crystal is shown. This signal (black symbols) is related to a shift-current-induced strain wave traveling through the piezoelectric material.^21^ Figure 6(b) includes two transients calculated for an x-ray source with purely Poissonian (blue symbols) or chaotic characteristics (red symbols) of the same average x-ray flux as the experimental source per delay point. The time dependence of Δ*I*/*I* and the average error of the experiment and the Poisson simulation are similar, and a SNR of roughly 40 is reached at a delay time of 300 ps [Fig. 6(c)]. This finding points to a predominant Poissonian character of the x-ray source. The ratio ΔSSNR is roughly 0.9, indicating that most temporal fluctuations of the femtosecond x-ray source are successfully compensated by normalization. In the case of a fully chaotic light source [red symbols in Fig. 6(b)], the average error is substantially increased with a SNR of only 20 [Fig. 6(c)]. The importance of a proper normalization is drastically illustrated in Fig. 6(b), where the orange symbols represent a transient without normalization by chopping. In this case, the average error for each delay point drastically increases by a factor of 10, leading to a maximal SNR of only 4 and rendering the transient change in diffracted intensity barely distinguishable from a case in which the shot-noise of a Poisson-distributed x-ray source is measured.

**FIG. 7.**
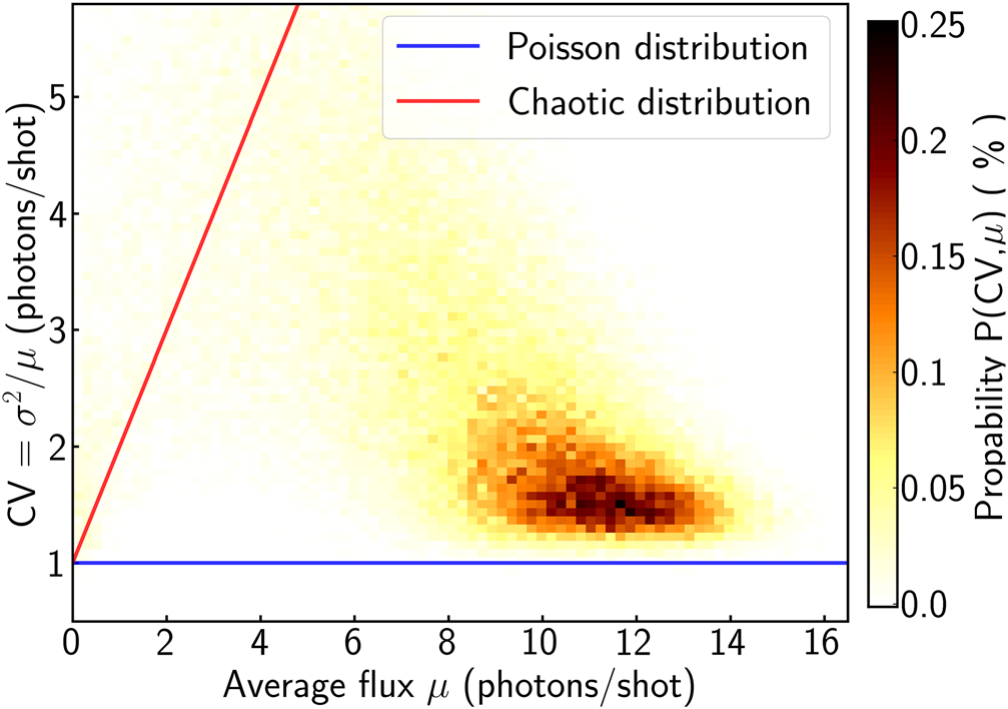
The photon-distribution width from a femtosecond x-ray diffraction experiment in comparison to the Poisson and chaotic light distribution functions. The modified coefficient of variation CV, i.e., the ratio of variance *σ*^2^ to expectation value *μ* is plotted as a function of the average flux *μ*. A total of 1.4 × 10^7^ shots measured on the (114) Bragg-reflection of LiNbO_3_ was partitioned in subsets with 250 consecutive x-ray shots. Their two-dimensional histogram is shown in a color coded way. For the Poisson case, CV is 1 (blue line), and for the chaotic light distribution, the relation $\text{CV}=(1+\bar{n})$ holds.

A behavior of our femtosecond x-ray source close to the Poissonian limit is also evident from an analysis of the statistical variation of the x-ray flux. In Fig. 7, the probability distribution of a modified coefficient of variation (CV = *σ*^2^/*μ*) for the (114) Bragg-reflection of LiNbO_3_ is shown as a function of the average flux *μ*. For a shot-noise limited light source ($\mu =\bar{n}$ and $\sigma^{2}=\bar{n}$), one obtains CV = 1 independent of the average flux per shot, illustrated by the blue line in Fig. 7. If the photon distribution deviates from the Poisson-statistics in the presence of additional source fluctuations, the variance of the photon-distribution is increased and results in CV > 1. For the purely chaotic x-ray source ($\mu =\bar{n}$ and $\sigma^{2}={\bar{n}}^{2}+\bar{n}$), CV is proportional to $\bar{n}+1$, as indicated by the red line. To determine the experimental distribution of CV as a function of *μ*, a total of 1.4 × 10^7^ measured shots was partitioned in subsets with 250 consecutive events, and CV and *μ* were calculated for each subset. The resulting two-dimensional histogram shows a distinct clustering of a majority of the points close to the shot-noise limit (CV = 1). In the absence of long term temporal fluctuations, the femtosecond x-ray source closely resembles an ideal Poisson light source, and shot-to-shot based fluctuations are of minor relevance.

## IV. CONCLUSIONS

In conclusion, we have analyzed the photon statistics and intensity fluctuations of a femtosecond laser-driven hard x-ray source by comparing experimental results with two limiting cases, a Poissonian and a chaotic light source. The experimental noise spectrum gives insights into the predominant mechanisms which are behind the fluctuations. The latter mainly originate from mechanical imperfections of the experiments and fluctuations of the driver laser. We demonstrate an experimental concept based on mechanical chopping of the optical pump pulses in an optical pump/x-ray probe experiment which eliminates temporal fluctuations of the measured signal efficiently and allows for experiments with a high sensitivity close to the shot-noise limit. Our analysis shows that a combination of femtosecond high-repetition laser drivers providing a high average optical flux with a detection scheme at the few-photon level provides the highest detection sensitivity of pump-probe experiments. Ongoing research and development aims at implementing this strategy with the help of femtosecond mid-infrared driver systems.
